# Determining the optimal choice of attenuation filters and propagation distance for polychromatic phase-contrast micro-computed tomography of a multi-material electromotor using synchrotron radiation

**DOI:** 10.1107/S1600577525002814

**Published:** 2025-04-23

**Authors:** Matthias Diez, Simon Zabler

**Affiliations:** a Chair of X-ray Microsopy LRM, Faculty of Physics and Astronomy University of Würzburg Josef-Martin-Weg 63 97074Würzburg Germany; b Deggendorf Institute of Technology DIT Dieter-Görlitz-Platz 2 94469Deggendorf Germany; University of Malaga, Spain

**Keywords:** synchrotron imaging, micro-computed tomography, signal-to-noise ratio, modulation transfer, MTF, image quality

## Abstract

Optimizing phase-contrast micro-computed tomography (µCT) for a given object is not trivial if the radiation is polychromatic and the object multi-material. This study demonstrates how an optimal combination of propagation distance and mean energy (set by attenuation filters) may be derived for such an object (an electromotor scanned on beamline BM18 at ESRF in Grenoble, France). In addition to appropriate image quality metrics, it is mandatory to define a task. In that respect, raising *E*_mean_ from 100 keV to 164 keV mitigates beam hardening by metal parts, yet raising *E*_mean_ further to 230 keV deteriorates CNR^2^ (where CNR is contrast-to-noise ratio) due to higher image noise. Propagation distances between *d* = 2 m and 25.3 m are evaluated crosswise with energy. While longer propagation distances generally yield higher CNR^2^, shorter distances appear favorable when discerning plastic near metal parts. SNR^2^ (where SNR is signal-to-noise ratio) power spectra and modulation transfer (MTF) are evaluated independently from two-dimensional projections supporting volume image analysis for which image sharpness depends strongly on the digital filters (Paganin and Wiener) which are applied along with filtered back-projection. In summary, optimizing synchrotron µCT scans remains a very complex task which differs from object to object. A physically accurate model of the complete imaging process may not only allow for optimization by simulation but also ideally improve CT image reconstruction in the near future.

## Abstract 2

Optimizing phase-contrast micro-computed tomography for a given object is not trivial if the radiation is polychromatic and the object multi-material. This study demonstrates how an optimal combination of propagation distance and mean energy may be derived for such an object – an electromotor scanned on beamline BM18 at ESRF, France.

## 1. Introduction

X-ray micro-computed tomography (µCT) started to become feasible at synchrotron light sources as well as with laboratory-CT scanners in the 1990s (Thompson *et al.*, 1984 ▸). While laboratory-CT uses micro-focal anodes, synchrotron µCT uses high-resolution pixel detectors for recording microscopic X-ray images (Stampanoni *et al.*, 2002 ▸).

Until today, those synchrotron pixel detectors have featured crystalline scintillator screens whose thickness and material determine (i) the detector’s resolving power and (ii) its detective efficiency with respect to the X-ray quanta. The latter are converted into a burst of optical photons whose intensity scales with the X-ray beam’s energy. A lens-coupled CMOS camera takes photographs of the back of the screen. These devices are generally referred to as lens-coupled indirect detectors (LCIDs) (Kim *et al.*, 2005 ▸).

The superiority of synchrotron µCT over laboratory µCT was established immediately after its introduction. The former features a superior contrast-to-noise ratio (CNR) thanks to propagation-based phase contrast (PBPC) (Zabler *et al.*, 2005 ▸). The extremely low divergence of synchrotron light sources allows for extending source–object distances (SOD) up to several hundred metres. In these settings, allowing optical propagation over several metres requires only distancing the object and detector over a similar distance (*d* ≃ ODD; ODD is the object–detector distance). Meanwhile, laboratory-based µCT takes place in cone-beam settings which do not allow more than few centimetres propagation distance *d*. This is because the propagation distance *d* = ODD/*M* is the normalized ODD with respect to the object magnification*M* = (ODD + SOD)/SOD.

Unlike laboratory-CT, few results have been published to optimize the experimental settings of synchrotron µCT (Saeid nezhad *et al.*, 2022 ▸). Beamline scientists operating µCT beamlines have implemented various beam optimization and instrument alignment protocols, each with different levels of automation. The optimal working point for X-ray imaging experiments is determined by the properties of each component of the experiment (*e.g.*X-ray beam, object and X-ray detector). The specifics of each component are defined by a set of experimental parameters.

The X-ray beam is defined by the spectral emittance of the X-ray (synchrotron) light source and the X-ray optics (including attenuation filters), and by its collimation with a series of two-dimensional slits. Most importantly for beamline BM18, the attenuation filters define the lower bound of the X-ray spectrum whereas the upper bound is fixed by the source. Note that, for low-energy applications, *K*-edge transmission through thin metal foils is also used as a low-energy filter.

The object is considered in the thin-lens approximation, a spatially distributed complex transmission function *T*(*x*, *y*) defined by the ray-projected sums of the object’s refractive index *n*, 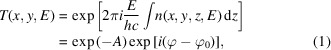
with *n*(*x*, *y*, *z*, *E*) = 1 − δ + *i*β the material’s refractive index, φ_0_ = 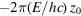, φ = 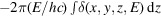 and *A* = 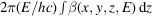 (Paganin, 2013 ▸). Here, the transmission is considered for monochromatic radiation of energy *E*, keeping in mind that polychromatic transmission is the sum over the entire spectrum of photon energies. *z*_0_ is the object thickness in the beam direction.

To introduce Fresnel propagation at a certain distance *z* = *d*, *T* is generally multiplied by an incoming plane wave Ψ_pw_(*x*, *y*, *E*) and the resulting product is convoluted with the two-dimensional Fresnel kernel *p*_*d*_(*x*, *y*, *E*),
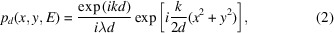
where *d* is called the *propagation distance*, approximately equating the ODD. Energy *E* is replaced by wavelength λ or wavenumber *k* = 2π/λ. Since both terms *n* and *p*_*d*_ depend on the photon energy, it is practical to consider the polychromatic spectrum of the synchrotron beam as a sum of quasi-monochromatic beamlets, and the recorded detector image as a sum of monochromatic images each representing the squared modulus (intensity) of a propagated wave,
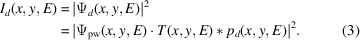
Note that for *d* = 0 (contact image) within this model the intensity equals the object’s transmission: *I*_*d*=0_(*x*, *y*, *E*) ∝ exp[−2*A*(*x*, *y*, *E*)].

For every energy *E*, the detector has different photon conversion and collection efficiencies expressed in energy-dependent weights *w*(*E*). Therefore the detected image is the weighted sum of the monochromatic beamlets,
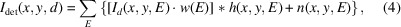
with *w*(*E*) = 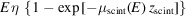. These weights take into account the energy integration by the scintillator screen, *i.e.* the product of its absorbance, the integral conversion efficiency η and the weighting by the X-ray energy *E*. We further assume that each beamlet has its own point spread function (PSF, *i.e.* joint pixel blur by source and detector) *h*(*x*, *y*, *E*), as well as its own share of noise *n*(*x*, *y*, *E*) (photon noise and electronic noise). Converting X-rays to optical photons and counting the latter results in correlated pixel noise, hence the dependency of *n* on the pixel coordinates (*x*, *y*). While there is only a limited choice of materials (this study uses an LuAG:Ce crystalline scintillator of *z*_scint_ = 2 mm thickness), the thickness of the screen can be defined to yield a compromise between spatial resolution and X-ray stopping power.

The image formation mentioned above allows for optimizing image quality by changing distinct experimental parameters. This study considers changing attenuation filters to alter the spectral properties of the X-ray beam, as well as changing the ODD which alters both *h*(*x*, *y*, *E*) and *p*_*d*_(*x*, *y*, *E*). While the former aims to optimize the polychromatic object transmission, the latter trades phase contrast (*i.e.* signal gain) for image blur (therefore the values of *d* remain small enough to avoid strong blur). Each parameter is known to feature an optimum individually. For the first time, we will consider optimizing both parameters jointly by recording a discrete set of µCT images on a two-dimensional parameter grid and evaluating their image quality on a multi-axis scale.

Saeid nezhad and co-workers recently succeeded in optimizing polychromatic object transmission in laboratory cone-beam setups, mainly by changing the upper energy bound of the X-ray spectrum through setting the X-ray anode voltage, the lower bound being defined by the object density and thickness (including attenuation filters) (Saeid nezhad *et al.*, 2022 ▸). The present study relies on changing the lower bound of the X-ray spectrum, so the image quality must be assessed differently.

On the one hand, insufficient filtering of the X-ray spectrum causes image artefacts to obscure structural information of the object. The test object for this study, a small electric motor, displays streak-shaped artefacts emerging at and tangential to strongly contrasted material interfaces (*e.g.* a straight metal–air interface). These streaks resemble metal artefacts (MA) which are frequently found in attenuation-contrast CT of multi-material objects. While MA in laboratory-CT are caused by very strong pixel-wise differences in transmission commonly occurring at material interfaces, similar effects are known to arise from likewise strong phase contrast differences in the same regions. Any deviation from the assumption that phase contrast equals a linear filter applied to the object’s transmission [equation (3)] will appear most visibly at these material interfaces and thus contribute to streak artefacts.

Meanwhile, beam hardening (BH) is superposed on MA, causing artificial density gradients inside metallic parts and a diffuse bright glow around the latter. Unlike streak artefacts, BH is mitigated by adding attenuation filters, but adding too much filter material will severely increase the measurement time, or – for constant exposure time – yield a worse signal-to-noise ratio (SNR). The latter suffers twofold from adding filter material: (i) through the loss of net photon flux and (ii) through the energy-dependent drop in material contrast (even though phase is less sensitive than attenuation in this respect).

Concerning ODD, the near-field condition sets the maximum propagation distance to *d*_max_ = 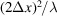, with Δ*x* the detector resolution (Weitkamp *et al.*, 2011 ▸). Respecting this limit is highly recommended for applying Paganin-type deconvolution to the polychromatic phase contrast in*I*_*d*_(*x*, *y*, *E*) [equation (3)]. While *d*_max_ is readily calculated for monochromatic beamlets, it can only be determined exactly by experiment in the case of polychromatic imaging of multi-material objects. The parameter *d*_max_ further defines the point beyond which material contrast cannot be increased by further extending the ODD given the cyclic property of the complex Fresnel kernel [equation 2]. In practice, the above-mentioned streak artefacts invite a reduction in the ODD well below *d*_max_ to keep these errors in check.

Note that biological objects (soft tissue, organs and small animals) generally have a low material contrast compared to electrical devices. It can therefore be advantageous to use long propagation distances up to *d*_max_ (matching *E*_mean_, see below) or even beyond. Here, even a loss in terms of resolution can justify an increase in contrast. Such objects generally lack the above-mentioned features, such as strong rectilinear material interfaces (Walsh *et al.*, 2021 ▸).

## 2. Materials and method

### 2.1. Experimental setup

The BM18 beamline is one of the latest synchrotron phase-contrast micro-tomography stations at the European Synchrotron Radiation Facility (ESRF), Grenoble. A three-pole wiggler source (3PW) delivers a powerful polychromatic beam with energies up to 350 keV and an X-ray footprint of 17 mm × 350 mm (vertical × horizontal) at the exit window 172 m downstream of the 3PW. The long experimental hutch allows the detectors to move continuously between ODDs ranging from 0 m to 36 m. By extending the ODD, poly­chromatic phase contrast can be efficiently used even at high photon energies and moderate pixel sizes. For phase retrieval by image post-processing, BM18 uses Paganin deconvolution, which is state of the art for polychromatic phase contrast images (Paganin *et al.*, 2002 ▸). It is generally applied two-dimensionally on X-ray images prior to back-projection and combined with a two-dimensional unsharp filter to compensate the blurring induced by the Paganin filter. This study, however, applies a double-pass deconvolution as well as the unsharp filter in three dimensions to filtered and back-projected volume images to account for the multi-materiality of the object. We compare µCT scans which were recorded at different detected mean energies *E*_mean_ (100 keV, 164 keV and 230 keV) and at different distances *d*. *E*_mean_ is altered by setting increasingly strong attenuation filters. The term ‘mean energy’ refers to the average photon energy of the detected photons. Summing over the spatial coordinates (*x*, *y*) in equation (4) yields a spatially averaged energy-dependent flat-field intensity *I*(*E*) = 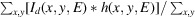 which allows calculation of the mean energy as the first normalized moment,
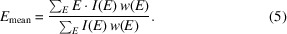
In order to evaluate experimentally the critical distance for propagation-based phase contrast for the test object, µCT scans were recorded at increasing ODD, placing the detector 2 m, 4 m, 16 m and 29.7 m downstream of the object.

**Figure 1:**
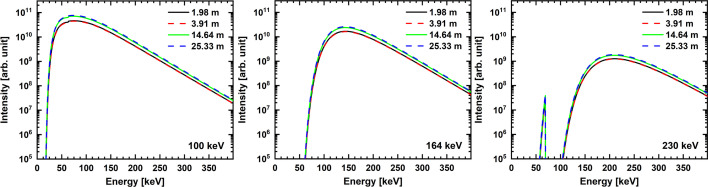
Semi-log graphs showing simulations of detected BM18 X-ray spectra. *E*_mean_ = 100 keV, 164 keV and 230 keV. Each graph shows spectra for 1.98 m, 3.91 m, 14.64 m and 25.33 m ODD and the filters from Table 1 ▸ including X-ray attenuation by air. *E*_mean_ is the mean energy for the central beam cross section of 37 mm × 11.84 mm, hence the detector field of view (FoV).

Table 1 ▸ lists attenuation filters which were chosen to yield different values of *E*_mean_. Note that for *d* shorter than 25.3 m the filter thickness of glassy carbon is increased to compensate for the lower absorption by air. The resulting detected spectra were simulated with the software *SPECTRA* (Tanaka & Kitamura, 2001 ▸) and are displayed in Fig. 1 ▸. All images were acquired during 16-bunch filling mode that results in a maximum beam current in the storage ring of 75 mA on beamline BM18 at the ESRF.

The X-ray camera is a lens-coupled indirect detector (LCID) with a 2 mm thick LuAG:Ce crystalline converter screen (Crytur, https://www.crytur.com/materials/luagce/). This LCID comprises a single 120 mm Hasselblad macro lens with an adjustable screen distance, allowing for optical sampling of the screen with a pixel size ranging from 4.25 µm to 14 µm (the former being the physical pixel size of the IRIS-15 sCMOS). The back of the screen is coated with an anti-reflective (AR) layer, reducing internal reflectance below 0.5% (535 nm wavelength). The AR layer was applied with electron-beam physical vapour deposition (EB-PVD). Both front and rear are polished to a smoothness of 5–6 nm. The screen’s front facing the X-rays is covered by 100–150 nm aluminium (reflective coating) applied with physical vapour deposition (PVD), topped by 80–100 nm SiO_2_ (protective layer) applied by EB-PVD. Aluminium shows a reflectance >90% at 535 nm wavelength; we recently reported a significant benefit in terms of SNR with respect to uncoated screens (Diez *et al.*, 2024 ▸). The lens projects the X-ray images from the screen onto an IRIS-15 sCMOS camera by Photometrics (Crytur, https://www.photometrics.com/products/iris-family/iris15). Prior to µCT, two-dimensional image series were recorded to determine the SNR^2^ power spectra (PS) amplitudes and the modulation transfer (MTF) at *E*_mean_ = 100 keV and for increasing propagation distances ranging from 1.98 m to 25.3 m (Table 1 ▸). For polychromatic imaging above 100 keV the screen’s MTF is assumed to be energy-independent (Compton scattering being the dominant attenuation). With luminescence from the whole screen’s thickness composing the images and with penumbral blurring being negligible at *d* = 2 m, the MTF does not degrade when the spectrum is hardened by additional filters. In the case of the source of BM18 with a size of 61 µm × 15 µm, the finite focal spot model results in penumbral blur up to ρ = 8.8 µm (Yu *et al.*, 2024 ▸). SNR^2^ PS allow quantification of the signal gain by phase contrast and include the detrimental effect of penumbral blurring which occurs when the propagation distance *d* is increased. The effects of hardening the spectrum with attenuation filters are known and therefore not detailed in this study: SNR^2^ decreases due to lower material contrast and worse photon statistics. Meanwhile, average photon conversion increases with *E*_mean_. The object sampling was constantly set to 14.8 µm per pixel. Hence, increasing the geometric magnification along with *d* was compensated by adjusting the LCID’s screen sampling (to 15.11 µm for 3.9 m, 16.11 µm for 14.6 m and 17.25 µm for 25.3 m).

Following SNR^2^ and MTF analysis, multiple µCT scans of a small electromotor were recorded at increasing propagation distances and for *E*_mean_ = 100 keV, 164 keV and 230 keV.

### 2.2. SNR power spectra

Signal *S* (deterministic) and noise *N* (stochastic) are considered additive and uncorrelated contributions to the detected intensities *I*_det_ [equation (4)]. Their power spectra can be derived separately from the Fourier transform of the latter according to the Wiener–Khintchine theorem (Khintchine, 1934 ▸): 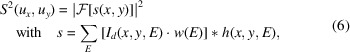

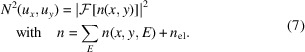
Note that *n*_el_ refers to pixel noise contributions stemming from electronic sources, *i.e.* readout and dark noise. The term SNR^2^ PS obviously refers to the ratio *S*^2^/*N*^2^ and it is derived from a series of *K* = 50 images. From the latter the average PS 〈*D*^2^〉 and the PS of the mean image intensity 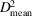 are computed, with *D*^2^(*u*_*x*_, *u*_*y*_) = 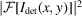, thus permitting calculation of SNR^2^: 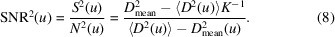
Here, for the sake of simplicity, the reciprocal coordinates (*u*_*x*_, *u*_*y*_) are radially averaged, becoming *u* = 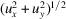. It is further important to distinguish SNR^2^ PS and temporal or pixel-wise SNR. For Poisson-distributed photon counts the latter scales with the square root of the number of photons Φ (quanta), whereas SNR^2^ scales linearly with Φ (and with exposure time accordingly) since it is a quadratic function. SNR^2^ furthermore includes the modulation transfer *H*^2^(*u*) and scales quadratically with the energy-dependent X-ray material contrast (attenuation and/or phase).

Sample images for SNR^2^ usually feature X-ray transmission by a homogeneous random structure, such as sandpaper or some other granular dispersion. Since this study uses relatively high X-ray energies for which sandpaper is completely transparent, a 4 mm thick pouch filled with copper chips was used instead for probing SNR^2^.

### 2.3. Modulation transfer function

The modulation transfer (MTF) was derived from images of a rectangular bar pattern which features on the MTF test phantom type 21 by Roentgen Huettner GmbH, on 0.03 mm thick structured lead foil. Unlike single-sided edge images whose Fourier transform (FT) (after spatial derivative across the edge) equals unity therefore sampling the MTF directly, the FT of the spatial derivative of a rectangle yields a sine. Hence, Fourier-transforming the derivative of a train of five rectangles equates the superposition of five sines. An MTF model fit can readily be applied to the maxima of the sines, whereby the superposition equates an effective pixel interpolation. The results are therefore equivalent to using a slanted edge (Illers *et al.*, 2005 ▸; Son *et al.*, 2014 ▸; Loot & Block, 2019 ▸). Having a relatively high transmission (∼20%) and a thin (0.03 mm) MTF phantom avoids BH and beam-alignment problems. The MTF is modelled here by the product of a Lorentzian with a Gaussian (Voigt fit): 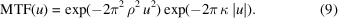
Note that analysing the MTF from images with strong phase contrast (*d* > 2 m) is problematic due to the entanglement of optical wave propagation and intensity convolution [equation (4)]. We will therefore only fit a Voigt model to the MTF measurements at *d* = 1.98 m, and discuss the MTF at longer propagation distances qualitatively.

Note that prior to Voigt fitting, the sampled MTF amplitudes are normalized to sinc(Δ*xu*) in order to remove the effect of rectangular pixel sampling.

### 2.4. Micro-CT scans

A small electric motor was chosen as a test object to find the optimal choice of attenuation filters and propagation distance. The motor comprises a salient pole rotor (steel wrapped into a coil of copper wire) inside a stator (radial arrangement of permanent magnets). The metallic parts are built into a plastic housing, making the motor a multi-material object. The critical challenge with such objects, from a CT imaging point of view, is the contrast visibility between air and plastic, particularly when the latter is in proximity to metallic parts. Contrast is expected to increase with propagation, whereas adding attenuation filters (to counter metal artefacts) will increase noise and reduce contrast. The squared contrast-to-noise ratio (CNR^2^) is an appropriate metric for evaluating image quality at the interfaces between plastic (*p*) and air (*a*),
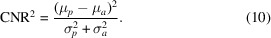
Here μ refers to the mean grey value in a defined image patch of one material and σ is the corresponding variance. As a quadratic metric CNR^2^ scales linearly with exposure time.

Each µCT volume image is built from 4000 projections acquired during 360° continuous object rotation, using filtered back projection by the inhouse software *NABU* (Paleo *et al.*, 2019 ▸). The scans at 100 keV accumulate 5 × 10 ms exposures for a single projection, whereas at 164 keV twice this time is used (5 × 20 ms) to compensate for the reduced photon flux. For the same reason 200 ms non-accumulated exposures constitute the 230 keV scans, hence twice the total exposure time compared with 164 keV (note that SNR^2^ and CNR^2^ are normalized with respect to total exposure time).

Phase retrieval by Paganin-type deconvolution is applied post CT-reconstruction as a two-pass filter using the *Pyxit* software package (Ullherr *et al.*, 2019 ▸; Ullherr & Zabler, 2015 ▸). Hereby, a volume mask is created for the strongly absorbing metal, then Paganin deconvolution is applied separately to the mask and to its complement using different parameters, thus avoiding blooming of the strong metal–air contrast into the remaining volume. In addition, a mask smoothing of size 4 × 4 is used to smooth out discontinuities at the edges of the mask. The Fourier filter kernel of each Paganin deconvolution is a Lorentzian,
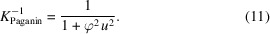
Note that 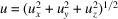 refers to three-dimensional reciprocal coordinates, and 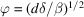 is a filter parameter referring to the material’s refractive index [equation (1)] and to the propagation distance (Weitkamp *et al.*, 2011 ▸). The filter parameters for metallic (*m*) and plastic (*p*) object parts hence depend on *E*_mean_ and *d*. Both φ_*m*_ and φ_*p*_ are listed in Table 2 ▸ for every scan. For defining the former, firstly optimal filter parameters were chosen manually for *d* = 14.64 m, *e.g.* yielding φ_*m*_ = 0.44 mm and φ_*p*_ = 0.75 mm for *E*_mean_ = 164 keV. Visual feedback allowed precise definition of these values so that no residual phase contrast (too low) and no artificial blooming (too high) would be observed outlining the corresponding material interfaces. Likewise, φ_*m*_ = 0.38 mm and φ_*m*_ = 0.62 mm were obtained for *E*_mean_ = 230 keV, and φ_*m*_ = 0.47 mm and φ_*m*_ = 0.92 mm for *E*_mean_ = 100 keV. Note that these values confirm that the phase contrast is increasing for lower energies. They are, furthermore, not far from the theoretical values,*e.g.* for 230 keV (mono-energy) the materials X-ray database (*XOP2.3*; Sanchez del Rio & Dejus, 2016 ▸) defines φ_14.64m,PMMA_ = 0.65 mm for plastic and φ_14.64m,Fe_ = 0.18 mm for iron. Secondly, all parameters for the remaining distances are calculated with φ_*d*_ = (*d*/14.64 mm)^1/2^ φ_14.64mm_.

Paganin-type phase retrieval corresponds to a low-pass filter blurring out the images. To revert this blur and for denoising, Wiener deconvolution is commonly applied in sequence with the Paganin filter, hence multiplication by an additional deconvolution filter kernel, 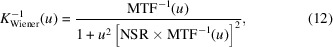
where NSR designates the noise-to-signal ratio, a scaling parameter inversely proportional to the temporal SNR. Note that, except for the inverse MTF MTF^−1^(*u*), 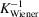 has the same form as 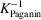.

For *d* = 14.64 m, manual parameterization yields ρ = 8.8 µm and κ = 5.92 µm, fixing these values for the remaining scans. The parameter NSR changes with *E*_mean_ due to differences in Φ, but does not scale with *d* whose effect is comprised in 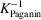. Therefore, the NSR is scaled manually for *d* = 14.64 m and the three values of *E*_mean_, yielding NSR = 0.048 for 230 keV, NSR = 0.039 for 164 keV and NSR = 0.027 for 100 keV, fixing these values for all other *d*.

## 3. Results

### 3.1. SNR power spectra

**Figure 2:**
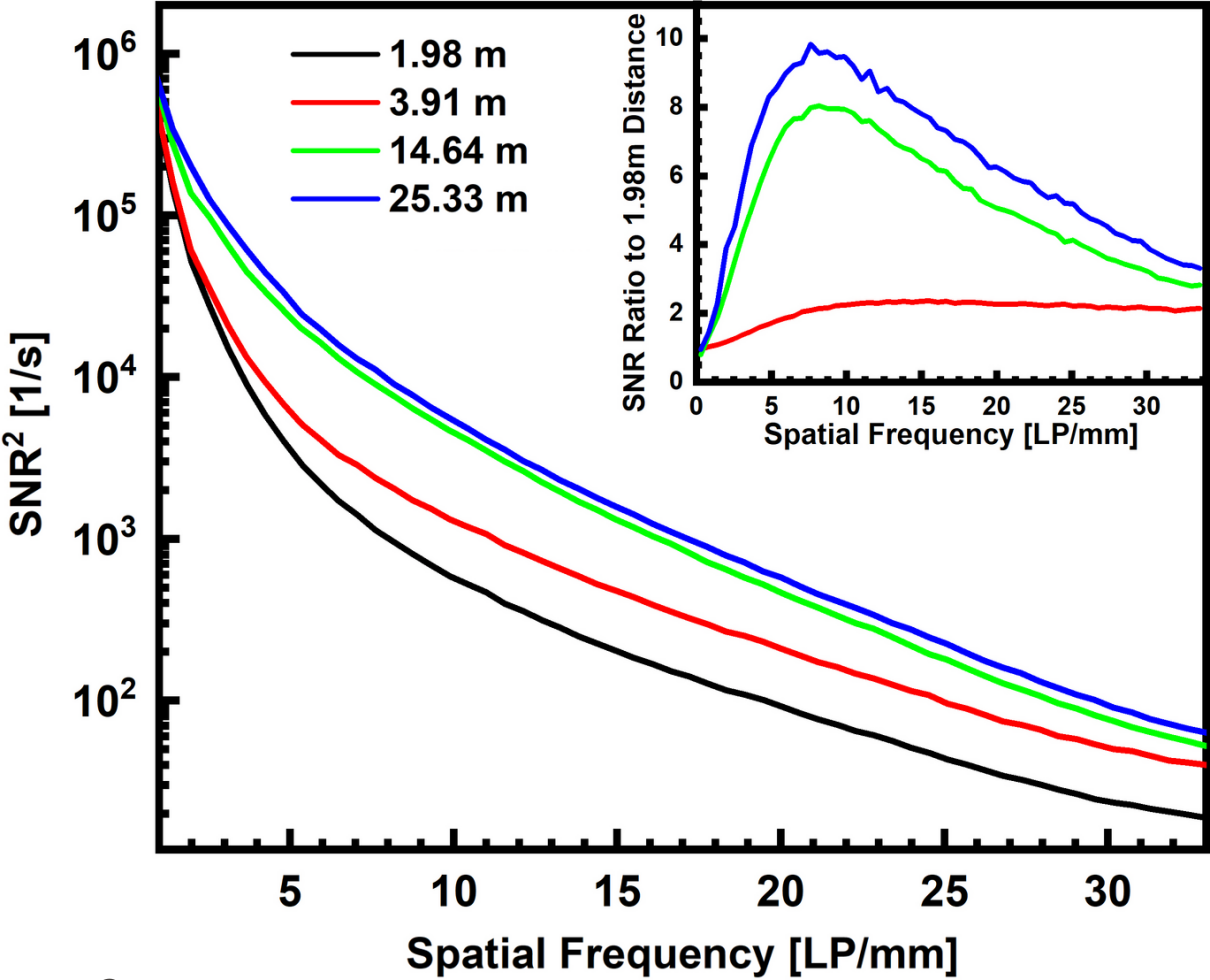
SNR^2^ PS of a pouch of copper flakes for *E*_mean_ = 100 keV for increasing propagation distances. The inset shows the frequency-dependent gain of PS amplitudes at 3.91 m, 14.64 m and 25.33 m with respect to *d* = 1.98 m.

The average transmission of the SNR^2^ phantom was 70.8 ± 8.9% at *d* = 2 m, 70.9 ± 9.4% at *d* = 3.9 m, 70 ± 12% at *d* = 14.6 m and 68 ± 13% at 25.3 m. Due to the lack of an optically denser phantom, SNR^2^ is only evaluated at 100 keV mean energy. The resulting PS are displayed in Fig. 2 ▸ for increasing propagation distance, along with the relative SNR^2^ gain with respect to the spectral amplitudes at *d* = 2 m. SNR^2^(*u*) is computed from 50 transmission, 50 flat-field and 50 dark images, according to equation (8). Meanwhile, a slightly increasing detector count is observed with increasing ODD. This increase is inferred to the attenuation differences between air (across the ODD) and carbon filters which are used for balancing the former. For the sake of comparability, all SNR^2^ spectra were therefore re-normalized to match the detector counts at *d* = 2 m.

Clearly, increasing the propagation distance results in a rise of SNR^2^ power for all non-zero spatial frequencies. The strongest gain is observed when *d* is increased from 3.9 m to 14.6 m. Increasing *d* further, *i.e.* from 14.6 m to 25.3 m, yields a visibly smaller gain. For the longer propagation distances (14.6 m and 25.3 m) the gain with respect to *d* = 2 m varies strongly with respect to spatial frequency. Unlike the gain at *d* = 3.9 m, these measurements feature peak gains at approximately 8 LP mm^−1^. Consequently, low frequencies are amplified more than high frequencies: at *u* = 10 LP mm^−1^ the gain at *d* = 3.9 m equals 2.2, at 16 m it is 7.9 and at 25.3 m it is 9.4, whereas for *u* = 20 LP mm^−1^ the gain is 2.2 at *d* = 3.9 m, 5.0 at 14.6 m and 6.2 at 25.3 m. At the Nyquist frequency, the SNR^2^ gain due to phase contrast is weakest, *i.e.* 2.1 at *d* = 3.9 m, 2.8 at 14.6 m and 3.3 at 25.3 m. Note that the strongest gain there still occurs for 25.3 m.

### 3.2. Modulation transfer function

**Figure 3:**
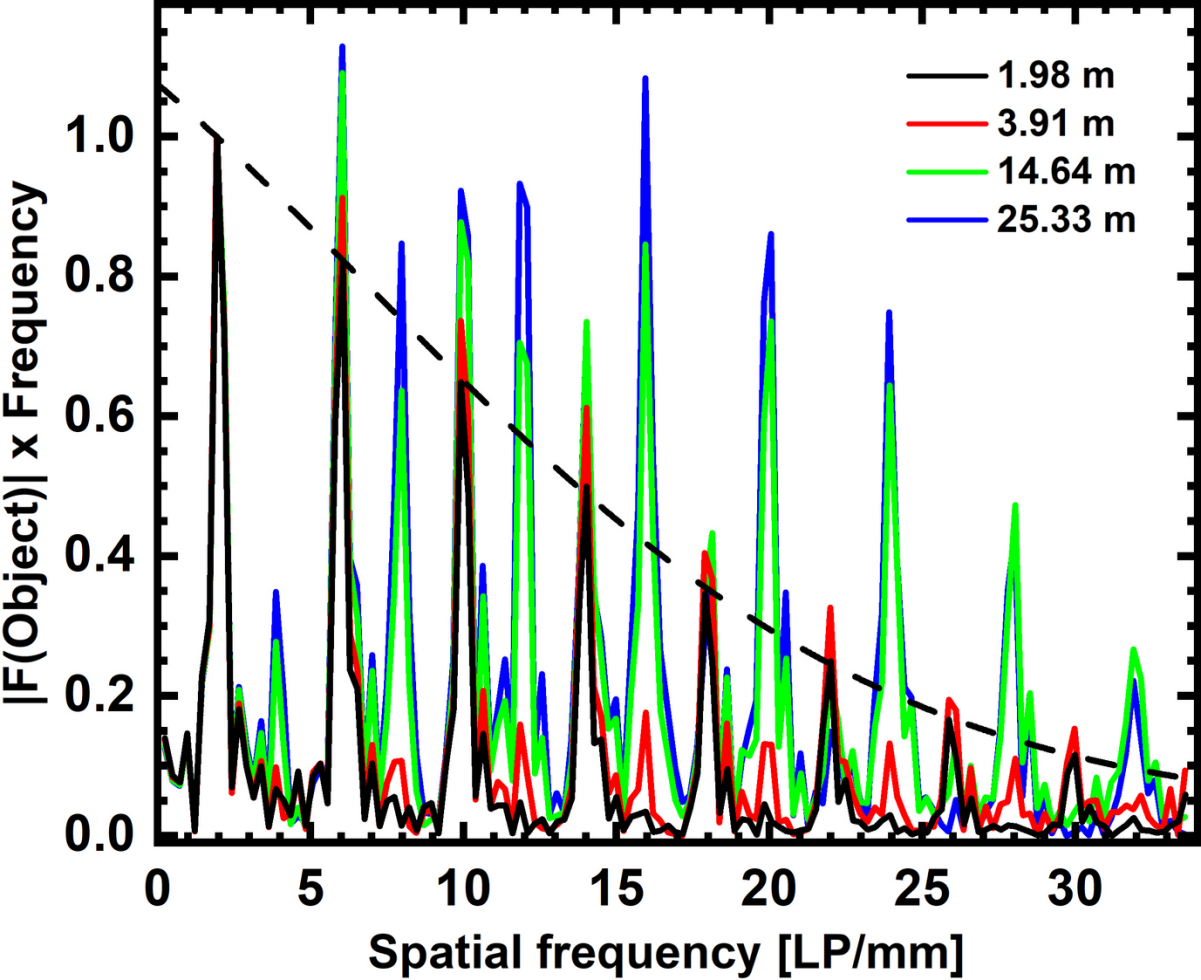
Frequency-weighted modulus of the bar pattern’s FT at *d* = 1.98 m, 3.91 m, 14.64 m and 25.33 m. The dashed line is the Voigt fit to the FT peaks at *d* = 1.98 m (image blur). All data were recorded for *E*_mean_ = 100 keV.

At *E*_mean_ = 100 keV, the average transmission of the MTF phantom, a 2 LP mm^−1^ bar pattern (five transparent bars in 0.03 mm lead), is 82.5 ± 1.1% at 2 m, 82.4 ± 1.1% at 3.9 m, 82.3 ± 1.2% at 14.6 m and 82.5 ± 1.1% at 25.3 m propagation distance. Fig. 3 ▸ shows the modulus of the Fourier amplitude of a line plot across the bar pattern at increasing propagation distances and after applying a Fourier derivative through multiplying the FT with *u*.

Note that these graphs comprise not just the MTF but also the object’s FT, as well as convolution of the latter with *P*_*d*_ [equation (3)]. Pure MTF amplitudes can therefore only be derived from the peaks in the Fourier spectrum of the 2 LP mm^−1^ bar pattern at *d* = 2 m.

At *d* = 2 m, only odd harmonics of the pattern’s FT appear (*i.e.* 2 LP mm^−1^, 6 LP mm^−1^, 10 LP mm^−1^*etc.*). For larger *d*, even harmonics (4 LP mm^−1^, 8 LP mm^−1^, 12 LP mm^−1^*etc.*) gradually appear in the spectrum with rising propagation distance. Increasing *d* up to 25.3 m continuously raises the power of the even harmonics, with the exception of the 16th harmonic which is strongest at *d* = 14.6 m.

Under the assumption that the measurement at *d* = 2 m represents quasi-pure absorption, it is appropriate to fit a Voigt function [equation (9)] to its spectral peaks to estimate the image blur. This fits yields ρ = 9.25 ± 0.92 µm and κ = 5.67 ± 0.61 µm. Note that within the parameters of the µCT scans (below), image blur is assumed to remain constant.

### 3.3. Micro-computed tomography

**Figure 4:**
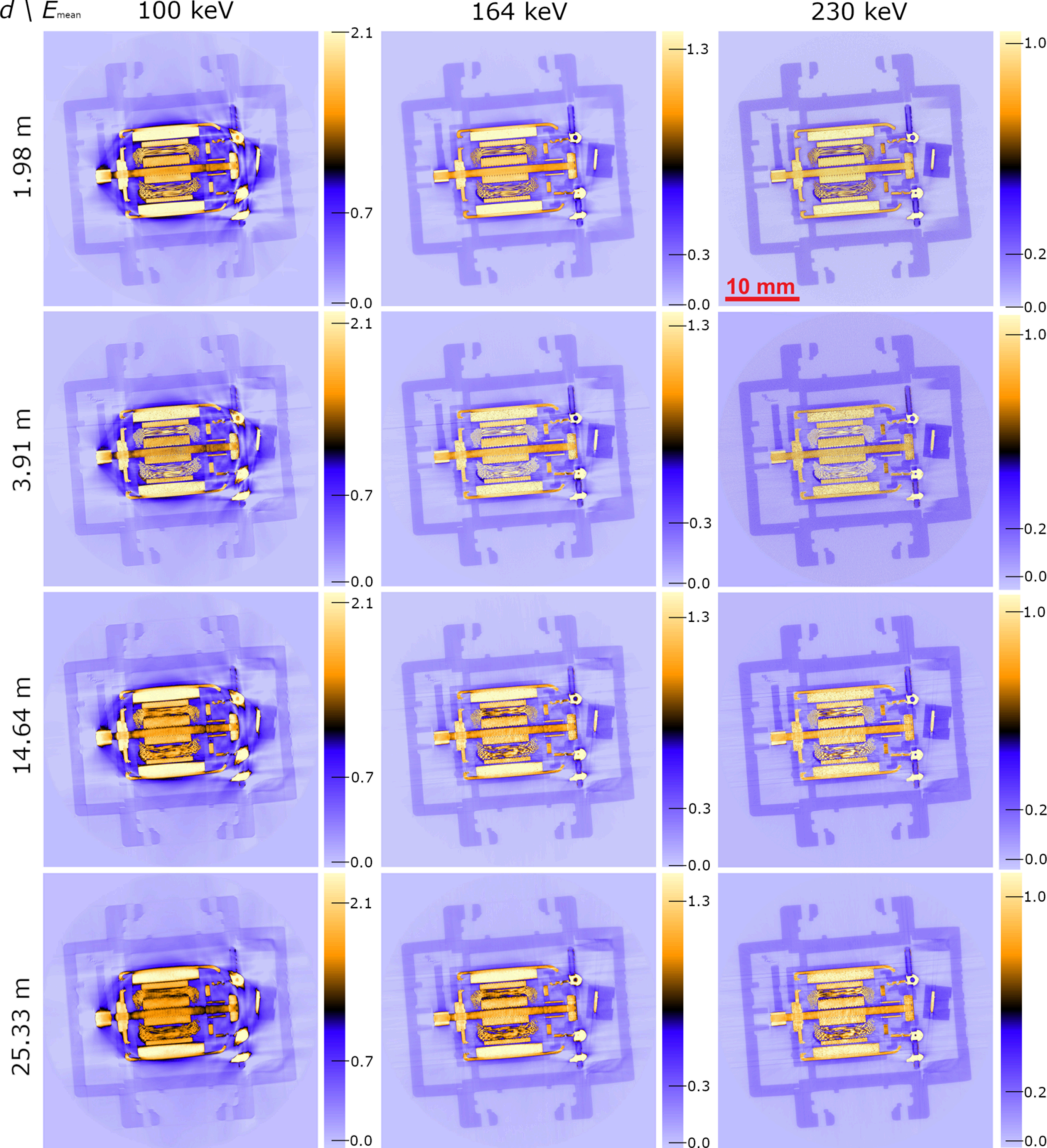
Axial slices from mµCT scans of a small electromotor at different propagation distances (rows) and at different mean energies (columns).The corresponding color maps are to the right of the images. The red bar in the top right-hand image measures 10 mm.

**Figure 5:**
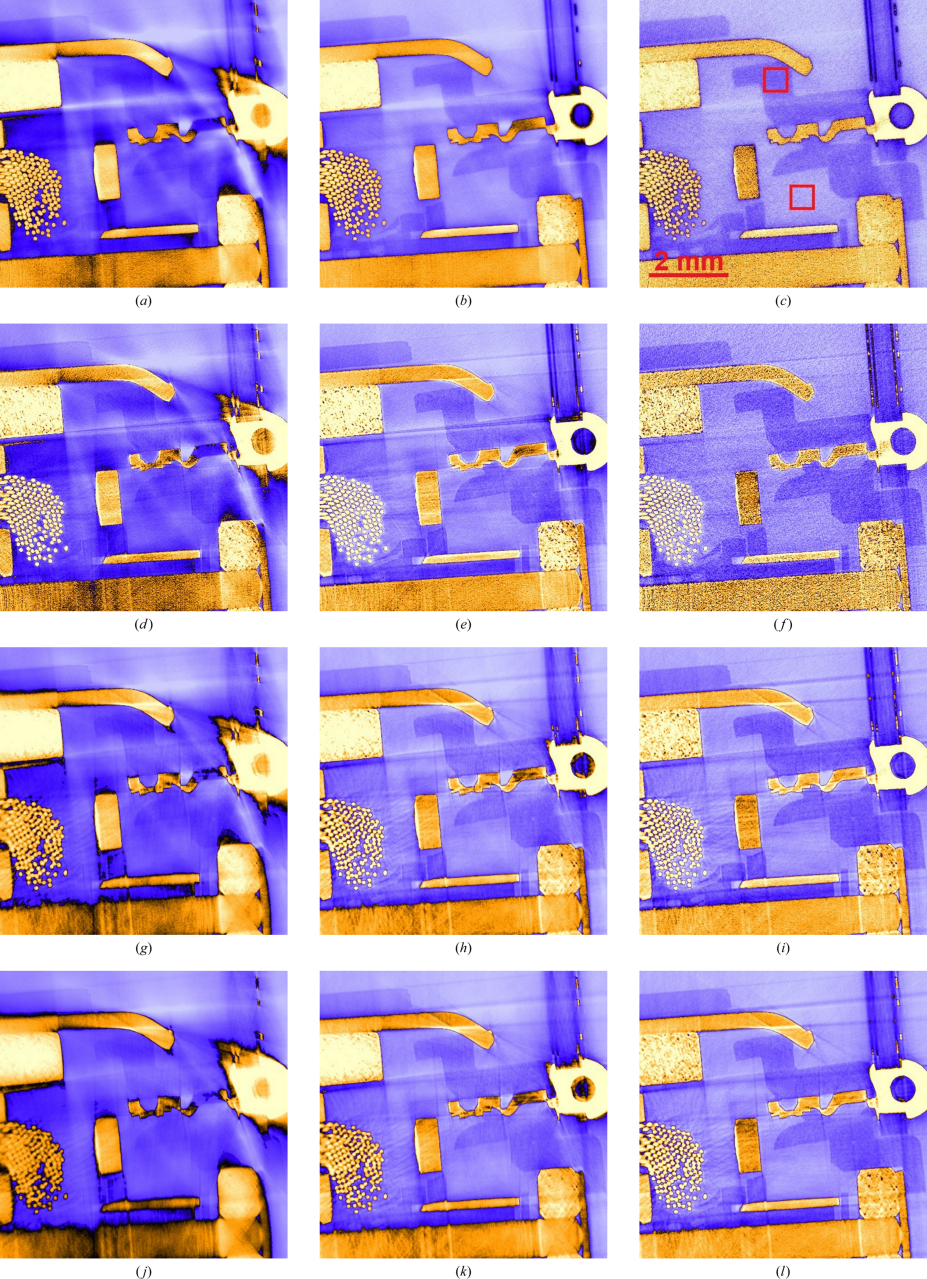
Enlargement of µCT sections showing the motor’s core with its metallic and plastic parts (*cf*. Fig. 4) at increasing propagation distance (rows) and mean energy (columns). The color map is the same as in Fig. 4. In the top right-hand frame, the two small red squares indicate measurements of the mean and standard deviation of attenuation in plastic and air. The red bar measures 2 mm.

A small electromotor (approximately 3 cm × 3 cm) serves as a test object for evaluating CT image quality at different propagation distances and at different mean energies, using the previously described experimental settings. Transmission through the motor’s metal piece, *i.e.* a 19.4 mm long metal rod as the most strongly attenuating part in the CT scan of this object, is on average 4.9% for 100 keV, 12% for 164 keV and 17% for 230 keV, with little variation for increasing propagation distance. Volume image reconstruction by filtered back-projection (FBP) is combined with two-pass (two-material) Paganin-type phase retrieval and Wiener deconvolution (filter parameters are listed in Table 2 ▸). Note that phase retrieval and deconvolution both apply post-reconstruction. Axial volume slices of the resulting reconstructions are shown in Fig. 4 ▸. For a more detailed view, these axial slices are magnified and cropped to the object’s centre where the metallic and plastic parts are closely packed (Fig. 5 ▸). To estimate the CNR^2^ between plastic and air in this area, the mean and standard deviation (STD) of both materials’ grey values are computed from the small boxes which are indicated in Fig. 5 ▸.

It is clearly visible in these images how BH decreases progressively from 100 keV to 230 keV mean energy. Meanwhile, the grey values of the plastic housing, which is further from the metal, is visibly less affected by BH. The BH also affects the grey values inside the metallic parts, causing artificial intensity gradients along directions of strong attenuation. Streak artefacts feature additionally in all scans, co-aligning with the directions of strong BH in metal. Note that while the 230 keV scans appear almost free of BH, streak artefacts remain although they are less pronounced. Particularly for 164 keV and 230 keV scans, these artefacts are more prominent and outlined by phase contrast for propagation distances of 3.9 m and above, compared with the scans at *d* = 2 m. Meanwhile, the SNR visibly degrades when *E*_mean_ is raised. The resulting pixel noise is most apparent in the transition from 164 keV to 230 keV for all propagation distances, but most strongly for *d* = 2 m and 3.9 m.

CNR^2^ for plastic and air were first evaluated near metallic parts (red boxes in Fig. 5 ▸) with the resulting values (STD, mean and CNR^2^) listed in Table 3 ▸. For comparison, these numbers were calculated for a second time (Table 4 ▸), this time in the plastic housing, which is further away from the metal and therefore much less affected by BH. Sufficiently far from metal parts (Table 4 ▸), the reconstructed attenuation of air approximates correctly to zero, except for the 100 keV scans which display a small offset (0.035). Meanwhile, the attenuation of plastic decreases steadily with mean energy (from 0.19 to 0.13) and so does the contrast (difference between the means). Both plastic attenuation and contrast remain un­affected by increasing propagation distance, at least within the error bounds of the measurement. Regarding CNR^2^ of plastic versus air, the results moderately increase from 100 keV to 164 keV (30–40%, except for *d* = 14.4 m where it is around 3%), then CNR^2^ drops sharply by a factor of ∼5 when the energy is raised to 230 keV. Regarding propagation distance, CNR^2^ increases for longer propagation distances, with the exception of *d* = 3.9 m for which CNR^2^ drops to ∼20–30% of its value at *d* = 2 m (for all energies). Yet at *d* = 25.3 m, CNR^2^ reaches 5–6 times its value for *d* = 2 m and 2.1 times its value for *d* = 14.6 m at 100 keV and 164 keV. This increase is even stronger for *E*_mean_ = 230 keV (12 times compared with *d* = 2 m and 3.2 times compared with 14.6 m).

When these results are re-examined but for plastic and air patches in proximity to metal (Table 3 ▸), some differences stand out. Plastic and air attenuation are both equally raised by artificial offsets due to BH. While this offset is ∼0.53 for air at 100 keV, this value drops to 0.15 and then to 0.07 for 164 keV and 230 keV, respectively. Judging from the results in Table 4 ▸, the offset for plastic is similar or even larger. This can be seen from the contrast, which is systematically 10–20% stronger than the corresponding values in Table 4 ▸. Meanwhile, the attenuation of both materials appears un­affected when the propagation distance is increased. Qualitatively, the CNR^2^ characteristics which are observed from Table 4 ▸ at least partly reproduce in the patches which are close to metallic parts. CNR^2^ increases from 100 keV to 164 keV. This increase is strongest for *d* = 2 m (4 times) and weakest for *d* = 14.6 m (1.4 times). For 230 keV CNR^2^ decreases to 32% of its former value at 164 keV. However, for *d* = 14.6 m and 25.3 m, CNR^2^ increases further for *E*_mean_ = 230 keV (by 39% and 53%, respectively). Regarding propagation distance, CNR^2^ decreases from *d* = 2 m to 3.9 m, then increases steadily for larger *d*. Only for *E*_mean_ = 164 keV and *d* = 25.3 m does CNR^2^ not surpass its initial value at *d* = 2 m which is exceptionally high (26.2). Meanwhile, all CNR^2^ values in Table 3 ▸ remain inferior to the corresponding values obtained from the plastic housing (Table 4 ▸). In turn, all standard deviations are significantly higher when measured near metallic parts.

In summary, the best CNR^2^ for plastic and air near metallic parts is found for *d* = 2 m and *E*_mean_ = 164 keV (CNR^2^ = 22.2), whereas further away from metallic parts *E*_mean_ = 164 keV displays by far the best CNR^2^ (584.7) at a propagation distance of *d* = 25.3 m.

**Figure 6:**
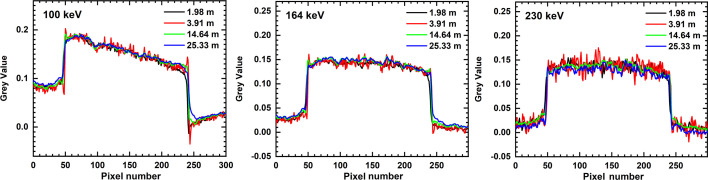
Line plots across the plastic housing (Table 4) showing attenuation values further away from metallic parts in the motor’s core at increasing mean energy (*cf*. Fig. 4).

For additional visual information, line profiles across a plastic part in the motor’s housing (*i.e.* far from metallic parts) are displayed in Fig. 6 ▸ for 100 keV, 164 keV and 230 keV mean energies. These profiles confirm the CNR^2^ values with those for *E*_mean_ = 164 keV, showing visibly less scattering for all propagation distances. BH appears as a linear gradient in the attenuation profiles for *E*_mean_ = 100 keV. This gradient remains noticeable but very faint at 164 keV, while it has completely disappeared at *E*_mean_ = 230 keV. In addition to these observations, the attenuation values at *d* = 3.9 m visibly scatter more than for all other distances. Note that at 100 keV and 164 keV, the line profiles at *d* = 3.9 m also display stronger residual phase contrast at the plastic–air interface.

## 4. Discussion

At *E*_mean_ = 100 keV, increasing the propagation distance step-wise up to 25.33 m yields higher SNR^2^ amplitudes up to the Nyquist frequency limit (here, 33 LP mm^−1^). While the present data testify to this relation qualitatively, the recorded SNR^2^ PS and the PS gain with respect to *d* = 2 m, in particular, display dependencies on spatial frequency as well as on propagation distance, which are not reproduced by a linear scaling in *d* alone. At first, the increase from *d* = 2 m to 3.9 m produces a gain of approximately 2.2 at 10 LP mm^−1^ and 7.9 when *d* increases to 14.6 m. Both values are relatively close to (7–8% larger than) the corresponding increase in propagation distance. However, when *d* is raised to 25.33 m the relative gain in SNR^2^ is only 9.4 (while 25.33 m/1.98 m ≃ 12.8). This observation is consistent with the assumption that phase contrast displays a sine-like (and not a linear) behaviour, saturating toward *d* = *z*_*c*_/2 ≃ 35 m (half the near-field limit, *i.e.* a maximum phase shift of π/2).

Meanwhile, for propagation distances larger than 3.9 m, the relative gain in SNR^2^ amplitudes weakens significantly for spatial frequencies larger than 10 LP mm^−1^. In these cases, the modulation transfer is approximately the product of a Laplacian gain *K*_Paganin_ with a strong image blur *H*, and is thus a peaked function which asymptotically approaches zero for high frequencies. When *d* is increased, *H* becomes steeper due to increasing penumbral blurring by the divergent X-ray source, hence the PS gain is further reduced for long distances and high frequencies. This phenomenon shows in the increasingly steep SNR^2^ PS, as well as in the reversal of the amplitude of the 16th harmonic of the MTF phantom (2 LP mm^−1^ bar pattern) which has a weaker amplitude at *d* = 25.3 m than at *d* = 14.6 m. Despite these observations, we abstained from changing *H* in 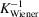. Such a change would require measuring *H* for all energies and for all propagation distances. MTF phantoms for higher energies would be required and the measurements would need to be matched to numerical simulations to take into account the effect of propagation.

The observed deviations from a linear scaling of modulation transfer with *d* have consequences for the parameterization of phase retrieval and Wiener deconvolution, both applied through Fourier filtering. In particular, the CT scan at *d* = 3.9 m displays higher pixel noise than all other scans. This effect can be countered by moderate changes in φ_*p*,*m*_, and thus by manually adapting Paganin’s kernel, which we avoided for the sake of comparability. Consequently, CNR^2^ is exceptionally weak for *d* = 3.9 m, with faint residual interference fringes outlining the plastic–air interfaces in the corresponding scans (Fig. 6 ▸).

Nevertheless, with the exception of *d* = 3.9 m, CNR^2^ increases steadily for higher propagation distances. Far from metallic parts its increase from *d* = 14.6 m to *d* = 25.3 m is thereby even more pronounced (twofold for 100 keV and 164 keV, threefold for *E*_mean_ = 230 keV) than what could be expected from SNR^2^ alone. For increasing *E*_mean_ one would expect CNR^2^ to drop, since the (phase/attenuation) contrast generally decreases with X-ray energy. However, raising *E*_mean_ from 100 keV to 164 keV results in a net increase in CNR^2^ for all propagation distances and equally whether the plastic is close to metal or not. Note that the parameter NSR in the Wiener deconvolution kernel was scaled with the square root of the estimated number of photons (mean flat-field intensity normalized to *E*_mean_) which was approximately twice as high for 100 keV than for 164 keV. Both photon statistics and energy-dependent contrast would therefore imply a drop in CNR^2^ from 100 keV to 164 keV, yet the contrary is observed. Indeed, material contrast does decrease but the STD of the grey values for air and plastic is much higher in the 100 keV scan. When the plastic is close to metal this difference in STD amounts to a factor of 2–3 with respect to *E*_mean_ = 164 kV, indicating that BH is responsible for the low CNR^2^ at *E*_mean_ = 100 keV. This assumption is corroborated by the far-reaching halo around the metal in Fig. 4 ▸, by the notably lower CNR^2^ (despite equal contrast) when the latter is evaluated near metallic parts, and by the observed contrast gradients in the corresponding line plots (Fig. 6 ▸). The decrease in CNR^2^ from 164 kV to 230 keV is in turn hardly influenced by BH and can be attributed to decreasing contrast and weaker photon statistics. Meanwhile, streak (metal) artefacts are present in all the scans, which is why CNR^2^ was evaluated in areas which appeared unaffected by these effects. Despite showing higher CNR^2^, the image sharpness at *d* = 25.3 m appears lower than at 14.6 m. While SNR^2^ measurements indicate that signal gain by phase contrast overcompensates this blur, reconstructed µCT slices give a different impression.

## 5. Conclusions

Optimizing phase contrast synchrotron µCT for an electromotor requires exact definitions of the object (multi-material, including metal parts) and the imaging task (*e.g.* accurately discerning plastic from air and metal). For choosing *E*_mean_, *i.e.* attenuation filters, focusing on the direction of highest attenuation is recommended. Analytically, a minimum of 11–12% transmission is required for monochromatic radiation, whereas polychromatic phase contrast imaging has higher optimal transmission (20–30%) to avoid strong BH and thus degradation of CNR^2^ (Graeff & Engelke, 2025 ▸). Regarding CNR^2^, *E*_mean_ = 164 keV was the best choice for resolving the plastic parts in the small electromotor. When comparing CNR^2^ for plastic parts distant from or close to metal parts, optimizing CNR^2^ is strongly affected by propagation distance. In particular, *d* = 2 m yields higher CNR^2^ near metal, whereas larger distances yield higher CNR^2^ far from metal. All scans at *E*_mean_ = 164 keV displayed residual BH in the plastic housing.

Note that this study discusses absolute values of CNR^2^ at different mean energies which are obtained from scans of different scan times (twice the scan time for 164 keV than for 100 keV, and again twice the scan time for 230 keV than for 164 keV). Since normalizing CNR^2^ with respect to scan time would always yield a preference for the lowest mean energy, despite strong BH, we compare scans of equivalent pixel count rates in order to discuss optimization with respect to BH. Summarizing, BH should be reduced independently of optimization of SNR^2^ and MTF. This study shows that such a combined approach would still yield a valid optimum for synchrotron scan parametrization.

For bigger or denser objects, realizing the above-mentioned transmission can be challenging, *i.e.* implying rather long measurement times due to increasing transparency of the scintillator for *E* > 200 keV and lost photon flux due to stronger filtering. Recent studies have further highlighted the importance of correcting intensity offsets, which are caused by diffuse light scattering inside the scintillator and which produce image artefacts similar to BH (Dremel & Fuchs, 2017 ▸; Hopkins, 2004 ▸). Correcting such artefacts remains an important task.

Concerning propagation, it appears safe to recommend values of *d* ≤ *z*_*c*_/2 (here, *z*_*c*_/2 = 35.4 m for 100 keV), at least for multi-material objects such as an electromotor. For a more detailed analysis it could be useful to re-evaluate *E*_mean_ while including the energy-dependent weighting of the (attenuation or phase) contrast, probably yielding lower values of *z*_*c*_ for *E*_mean_. While increasing the propagation distance always yields higher SNR^2^ PS amplitudes (estimated from two-dimensional projection images), the same is not necessarily true for CNR^2^. While the best CNR^2^ for plastic parts far from metal is found at the longest propagation distances, the best value for plastic in proximity to metal is found at *d* = 2 m. Meanwhile, spatial resolution, in terms of modulation transfer MTF, is accounted for in SNR^2^ but not in CNR^2^. In this study we abstained from estimating the MTF, *i.e.* the sharpness of the volume images. While two-dimensional measurements reveal that the combined effect of phase contrast and MTF improves with increasing propagation distance up to its highest value, the (volume) image processing chain which combines Paganin and Wiener deconvolution may strongly alter this effect. This study further revealed that scaling the parameters of both deconvolution kernels accordingly with *d*, *E*_mean_ and photon flux does not yield identical image sharpness and can even deteriorate CNR^2^. An insufficient scaling of φ_(*m*,*p*)_ with *d* is the most plausible explanation for the low CNR^2^ values at *d* = 3.9 m observed in this study. In conclusion, optimizing synchrotron µCT for a given object must include a careful evaluation of the employed filters and their parameters, particularly with respect to image sharpness and CNR^2^.

In summary, this study demonstrates how an optimal combination of propagation distance and mean energy (by choice of attenuation filters) can be found for a given object and imaging task. While this study considers the task of discerning plastic from air or from metal, alternative tasks could apply, for example finding material defects in a matrix or accurately reconstructing the surface of an object part may lead to different optimal settings. If quality metrics are chosen carefully, succeeding global optimization may even profit from supporting simulations which in turn require an accurate model of the imaging physics. This study revealed that using only one quality metric may be insufficient, *e.g.* since CNR^2^ does not account for loss of image sharpness. The latter can be evaluated independently from SNR^2^ or MTF.

Streak (metal) artefacts were observed but not treated in this study. Recent studies indicate that iterative combined schemes of CT back-projection and phase retrieval can overcome the limits imposed by linearizing phase contrast and hence correct second-order effects which may cause these artefacts (Ruhlandt & Salditt, 2016 ▸; Zhao *et al.*, 2018 ▸).

## Figures and Tables

**Table 1:** The attenuation filters for the different values of *E*_mean_ and propagation distances *d* Al_2_O_3_ refers to sapphire, C to glassy carbon, and Mo and W to metal plates of molybdenum and tungsten, respectively.

*d*/*E*_mean_	100 keV	164 keV	230 keV
1.98 m	Al_2_O_3_: 5 mm	Mo: 3.75 mm	W: 2 mm
	C: 134.5 mm	C: 60 mm	C: 45 mm
3.91 m	Al_2_O_3_: 5 mm	Mo: 3.75 mm	W: 2 mm
	C: 134.5 mm	C: 60 mm	C: 45 mm
14.64 m	Al_2_O_3_: 5 mm	Mo: 3.75 mm	W: 2 mm
	C: 104.5 mm	C: 30 mm	C: 15 mm
25.33 m	Al_2_O_3_: 5 mm	Mo: 3.75 mm	W: 2 mm
	C: 89.5 mm	C: 15 mm	

**Table 2:** The used Paganin parameters of the two-step Paganin-type post-processing phase retrieval for plastic (*p*) and metal (*m*)

Filter parameter		100 keV	164 keV	230 keV
1.98 m	φ_*p*_	0.34 mm	0.28 mm	0.23 mm
	φ_*m*_	0.17 mm	0.16 mm	0.14 mm
3.91 m	φ_*p*_	0.48 mm	0.39 mm	0.32 mm
	φ_*m*_	0.24 mm	0.23 mm	0.20 mm
14.64 m	φ_*p*_	0.92 mm	0.75 mm	0.62 mm
	φ_*m*_	0.47 mm	0.44 mm	0.38 mm
25.33 m	φ_*p*_	1.21 mm	0.99 mm	0.82 mm
	φ_*m*_	0.62 mm	0.58 mm	0.50 mm

**Table 3:** Mean attenuation (Mean) and standard deviation (Dev.) for plastic and air (in proximity to metallic parts, *cf*. Fig. 5) for increasing mean energy (100 kV, 164 kV and 230 keV) and propagation distance (1.98 m, 3.91 m 14.64 m and 25.33 m) Contrast is the difference of means. CNR^2^ is defined by equation (10).

Plastic (P) and Air (A) within the metal				
*d*		100 keV	164 keV	230 keV
1.98 m	Mean (P)	0.723	0.316	0.229
	Dev. (P)	0.058	0.024	0.035
	Mean (A)	0.520	0.146	0.081
	Dev. (A)	0.062	0.023	0.037
	Contrast	0.203	0.170	0.148
	CNR^2^	5.7	26.2	8.4
3.91 m	Mean (P)	0.712	0.303	0.228
	Dev. (P)	0.067	0.044	0.067
	Mean (A)	0.531	0.149	0.081
	Dev. (A)	0.064	0.036	0.069
	Contrast	0.181	0.154	0.147
	CNR^2^	3.8	7.3	2.3
14.64 m	Mean (P)	0.699	0.299	0.220
	Dev. (P)	0.045	0.040	0.030
	Mean (A)	0.528	0.146	0.069
	Dev. (A)	0.039	0.021	0.023
	Contrast	0.171	0.153	0.151
	CNR^2^	8.2	11.5	16.0
25.33 m	Mean (P)	0.687	0.290	0.221
	Dev. (P)	0.036	0.033	0.026
	Mean (A)	0.542	0.149	0.074
	Dev. (A)	0.032	0.018	0.018
	Contrast	0.145	0.141	0.147
	CNR^2^	9.1	14.1	21.6

**Table 4:** The determined mean value (Mean) and deviation (Dev.) of the grey values of plastic and air from the top right-hand part of Fig. 4, and the resulting contrast and CNR^2^

Plastic (P) and Air (A) outside the metal				
*d*		100 keV	164 keV	230 keV
1.98 m	Mean (P)	0.191	0.142	0.131
	Dev. (P)	0.013	0.010	0.030
	Mean (A)	0.035	0.005	0.004
	Dev. (A)	0.013	0.010	0.027
	Contrast	0.156	0.137	0.127
	CNR^2^	72.0	93.8	9.9
3.91 m	Mean (P)	0.190	0.143	0.132
	Dev. (P)	0.029	0.022	0.051
	Mean (A)	0.033	0.004	0.005
	Dev. (A)	0.027	0.020	0.055
	Contrast	0.157	0.139	0.127
	CNR^2^	15.7	21.9	2.9
14.64 m	Mean (P)	0.195	0.148	0.126
	Dev. (P)	0.008	0.007	0.015
	Mean (A)	0.035	0.007	-0.003
	Dev. (A)	0.007	0.006	0.015
	Contrast	0.160	0.141	0.129
	CNR^2^	226.5	233.9	37.0
25.33 m	Mean (P)	0.196	0.148	0.133
	Dev. (P)	0.006	0.005	0.009
	Mean (A)	0.036	0.007	0.003
	Dev. (A)	0.005	0.003	0.008
	Contrast	0.160	0.141	0.130
	CNR^2^	419.6	584.7	116.6

## Data Availability

Data underlying the results presented in this paper are not publicly available at this time but may be obtained from the authors upon reasonable request.
